# A review on cell damage, viability, and functionality during 3D bioprinting

**DOI:** 10.1186/s40779-022-00429-5

**Published:** 2022-12-16

**Authors:** He-Qi Xu, Jia-Chen Liu, Zheng-Yi Zhang, Chang-Xue Xu

**Affiliations:** 1 Department of Industrial, Manufacturing, and Systems Engineering, Texas Tech University, Lubbock, TX 79409 USA; 2 School of Naval Architecture and Ocean Engineering, Huazhong University of Science and Technology, Wuhan, 430074 China

**Keywords:** Three-dimensional bioprinting, Cell damage, Shear stress, Cell viability, Cell functionality

## Abstract

Three-dimensional (3D) bioprinting fabricates 3D functional tissues/organs by accurately depositing the bioink composed of the biological materials and living cells. Even though 3D bioprinting techniques have experienced significant advancement over the past decades, it remains challenging for 3D bioprinting to artificially fabricate functional tissues/organs with high post-printing cell viability and functionality since cells endure various types of stress during the bioprinting process. Generally, cell viability which is affected by several factors including the stress and the environmental factors, such as pH and temperature, is mainly determined by the magnitude and duration of the stress imposed on the cells with poorer cell viability under a higher stress and a longer duration condition. The maintenance of high cell viability especially for those vulnerable cells, such as stem cells which are more sensitive to multiple stresses, is a key initial step to ensure the functionality of the artificial tissues/organs. In addition, maintaining the pluripotency of the cells such as proliferation and differentiation abilities is also essential for the 3D-bioprinted tissues/organs to be similar to native tissues/organs. This review discusses various pathways triggering cell damage and the major factors affecting cell viability during different bioprinting processes, summarizes the studies on cell viabilities and functionalities in different bioprinting processes, and presents several potential approaches to protect cells from injuries to ensure high cell viability and functionality.

## Background

**Fig. 1:**
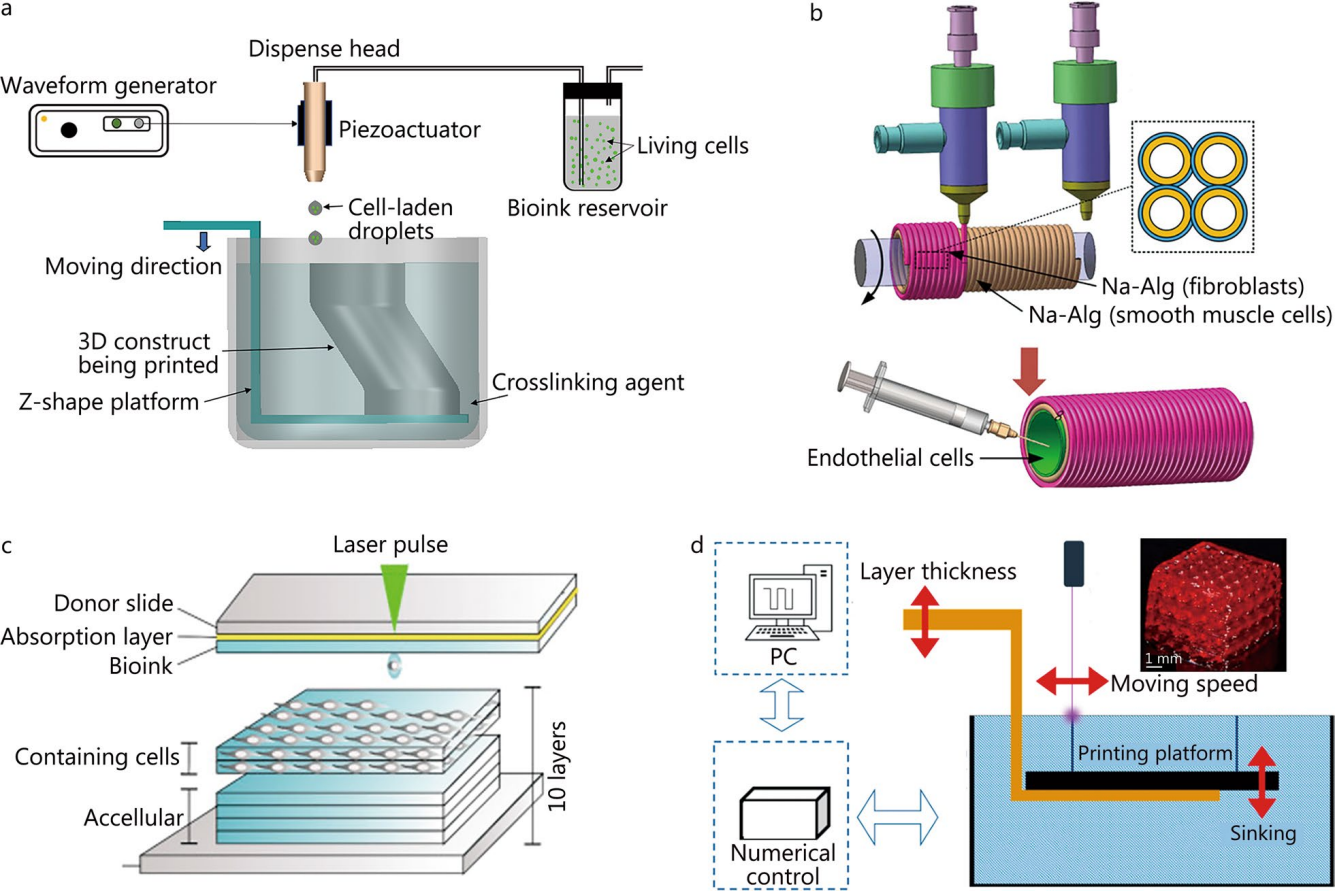
Schematic diagram of bioprinting technique. **a** Inkjet-based bioprinting. Reprinted with permission from Reference [8]. Copyright 2019, the American Institute of Physics. **b** Extrusion-based bioprinting. Na-Alg sodium alginate. Reprinted with permission from Reference [9]. Copyright 2017, ACS. **c** Laser-assisted bioprinting. Reprinted with permission from Reference [10]. Copyright 2018, Elsevier. **d** Stereolithography-based bioprinting. Reprinted with permission from Reference [11]. Copyright 2020, Elsevier

The demand for the transplantation of organs is consistently increasing due to the severe organ failure problem and shortage of suitable donors [1, 2]. Three-dimensional (3D) bioprinting which fabricates 3D functional tissues/organs (e.g., human skin [3]) by precisely positioning the bioink containing biological materials and living cells in a layer-by-layer manner has shown great promises for tissue engineering [4, 5]. Generally, dependent on the printing mechanisms, 3D bioprinting can be classified into four typical types, including inkjet-based bioprinting, extrusion-based bioprinting, laser-assisted bioprinting, and stereolithography-based bioprinting [6, 7]. The schematic diagram of each bioprinting technique is representatively shown in Fig. 1 [8–11]. Inkjet-based bioprinting usually relies on thermal expansion- or piezoelectric actuation-induced pressure to eject the cell-laden droplets out of the nozzle [12, 13]. Inkjet-based bioprinting has good controllability on the size and deposition of the cell-laden droplets during printing [14]. However, due to its limited nozzle size, bioink with high concentrations/viscosities can hardly be ejected out of the inkjet nozzle [15, 16]. As another type of nozzle-based bioprinting technique, extrusion-based bioprinting system can be driven by either a pneumatic pressure-, piston-, or solenoid-based system to dispense cell-laden bioink [17]. Extrusion-based bioprinting is favored for its ability to print bioink with high concentrations and viscosities despite its relatively poor printing resolution and low cell viability. On the basis of laser-induced forward transfer (LIFT) technique, laser-assisted bioprinting relies on the pressure bubble generated by the laser pulse to eject the suspended bioink [18]. Benefiting from its nozzle-free mechanism, laser-assisted bioprinting is leveraged to eliminate the challenges in nozzle-based bioprinting systems. It is not limited by the concentrations/viscosities of the bioink and is capable to form 3D constructs with high precision and cell viability. However, its further applications are restricted by its high cost and time-consuming process [19, 20]. Stereolithography-based bioprinting usually relies on ultraviolet (UV) light or visible light to crosslink photocrosslinkable materials in a layer-by-layer manner until the completion of the 3D complex structures [21, 22]. Stereolithography-based bioprinting can be further divided into two categories: conventional stereolithography where a light source is controlled with a point-by-point movement on each layer, and digital light processing (DLP) where a projector is used to project the light source onto each layer of photocrosslinkable materials [23]. Stereolithography-based bioprinting is capable to fabricate 3D constructs with complex geometries with high printing resolution, but the overall printing time is sometimes longer than other techniques due to its crosslinking mechanism, and the currently limited choices of materials possessing both biocompatibility and photocrosslinkable properties restrict its broader applications [24, 25].

3D bioprinting has a rigid requirement on the selection of materials [26]. Bioink, as a key element for 3D bioprinting, is a mixture of biological materials and the desired cell types [27]. Suitable biological materials should hold the attributes such as good biocompatibility and biodegradability, easy crosslinking mechanism, and robust mechanical properties, to name a few [28, 29]. Among all the materials, water-soluble polymers also known as hydrogels are currently found to be the most suitable biological materials for the bioink due to their similarities to natural extracellular matrix (ECM) enabling the adhesion, proliferation, and differentiation of the encapsulated living cells [30]. Generally, the commonly used hydrogels for 3D bioprinting can be classified into two typical categories including natural hydrogels (such as collagen [31], silk [32], alginate [33], and fibrin [34]) and synthetic hydrogels [such as polyethylene glycol (PEG) [35] and polyvinyl alcohol (PVA) [36]]. Even though natural hydrogels are proven to be more biocompatible compared to synthetic hydrogels, synthetic hydrogels can provide tunable mechanical and physical properties [37]. As the other key component of the bioink, various types of cells including primary cells, cell lines, and stem cells have been incorporated for 3D bioprinting. Primary cells refer to the cells which are directly isolated from tissues/organs, cell lines refer to the culture of cells originating from a primary cell culture, and stem cells hold the ability to develop into different cell types under guidance. For example, stem cells [such as mesenchymal stem cells (MSCs) [38] and embryonic stem cells (ESCs) [39]] and primary cells [such as human umbilical vein endothelial cells (HUVECs) [40] and 3T3 mouse fibroblasts [41, 42]] have been used as the model cells, mixed within the prepared hydrogel solution, and incorporated in various 3D bioprinting applications. With the advances in material science and broader choices for biological materials, 3D constructs such as vessel-like constructs [43], cartilage [44], bone [45], and skin [46] have been fabricated using various bioprinting techniques.

Recently, the continuous advancements in 3D bioprinting techniques and broader choices of suitable biological materials have enabled the rapid and precise biofabrication of 3D artificial tissues/organs such as skin [47] and bone [48]. As a rapidly developing technology, 3D bioprinting which has the potential to fabricate 3D native-like constructs with complex geometries has shown great promises for solving the severe organ failure problem and replacing the malfunctioned tissues/organs [49]. There are frequent injuries in the combat zone and the resultant blood vessel damage increases the probability of mortality [50]. Specifically, the muscle loss due to trauma is frequently observed on the battlefield, and it is extremely difficult for soldiers with injuries to receive immediate treatment such as surgery due to the harsh environment in the combat zone. The recovery of soldiers’ wounds and injuries remains challenging. Therefore, it remains critical to find an optimal solution to promptly and properly treat the wound in the remote combat zone [51]. The advance of 3D bioprinting provides a promising solution for promptly healing wounds caused on the battlefield [52]. For example, 3D bioprinting such as inkjet-based bioprinting can be chosen as an effective tool for the treatment of frequent traumas such as skin/bone injuries and blood vessel damage on the battlefield [53]. These 3D-bioprinted skins/bones are customizable and transplantable to the injured soldiers. 3D-bioprinted vessel-like constructs such as vascular grafts will be helpful for promptly and temporarily treating blood vessel damage severe bleeding on the battlefield before the patients are transferred for formal physical surgery [54]. In addition, 3D-bioprinted constructs which mimic native tissues/organs can be applied to other areas such as drug discovery and investigation of blast injuries. To conclude, with the continuous advancements in 3D bioprinting techniques and broader choices of materials, 3D bioprinting has a great potential to get involved in military-related fields for better healing combat-related injuries [55].

Even though 3D bioprinting techniques have experienced significant improvements during the past decade, maintaining high cell viability and functionality after the bioprinting processes remains challenging [56]. All these four 3D bioprinting techniques were found to adversely affect cell viability due to the stresses during the bioprinting process [57]. In addition, cell viability may further decrease while being cultured within a nutrition-deficient environment [58]. Generally, stresses generated during the bioprinting processes affect cell viability and functionality by influencing cell signaling and protein expression [59]. To reduce the percentage of cell death and ensure post-printing cell functionality, the overall stress must be carefully controlled. In brief, the stress imposed on the cells is either due to the shear stress generated from the imposed pressure and nozzle size during the nozzle-based bioprinting process [60, 61], or the thermal and radiative stress generated by the light sources during light-based printing processes [62]. For example, during extrusion-based bioprinting, the stress mainly comes from the shear stress, and the magnitude of the overall shear stress is determined by several parameters including the nozzle size, pressure, printing speed, and viscosity of the bioink [63]. The detailed information on the factors affecting cell viability during each type of bioprinting technique will be covered in the following sections.

This review discusses the factors resulting in cell damage during each type of bioprinting, and summarizes several typical studies on cell viability and functionality after different bioprinting processes. The aim of this review is to gain a better understanding of each type of 3D bioprinting by summarizing the mechanism of cell damage and explore possible ways to maintain high cell viability and functionality after the printing processes and thus achieve better printing performance.

## Cell damage during 3D bioprinting

Due to the complicated cellular behaviors, living cells are more complicated than other normal engineered materials (e.g., nano-particles) [64]. Therefore, it is necessary to understand the relationship between biological damage pathways and cell damage to manage the unwanted cell viability loss. During the bioprinting processes, cell injury may source from various inducements/stimuli such as shear stress, thermal stress, and radiative stress. The percentage of cell injury is dependent on the strength and duration of the stimuli. If the imposed stresses exceed the loading capacity of a single cell, irreversible cell damage will happen, resulting in unexpected cell death such as cell apoptosis. Due to the unique mechanism of each bioprinting technique, the factors affecting cell viability vary from each other. Briefly, during nozzle-based bioprinting such as inkjet-based bioprinting and extrusion-based bioprinting, cells are mainly experiencing shear stress during the ejection process [65], while during light-involved bioprinting such as laser-assist bioprinting and stereolithography-based bioprinting, cells mainly endure the thermal and radiative stress induced by different wavelengths of lights and the shear stress [66]. In addition to the shear stress, several other environmental factors such as temperature and pH may also affect cell viability. For example, temperature is able to affect the rheological properties and gelation of the bioink and thus affect cell viability [67], and pH is able to affect the hydrogel properties such as the pore size [68] and thus affect cell viability [69]. It is of great significance to understand the cell injury sources and model the cell viability during the bioprinting process. In this section, the main triggered pathways indicating cell damage/death, the typical factors affecting cell viability in each type of 3D bioprinting technique, and several representative modellings to predict cell damage and cell viability will be discussed and summarized.

### Cell damage pathways

Cell death is generally classified into apoptosis and necrosis [70]. Apoptosis, reflecting the fate of cells, refers to the malfunctions of the cells coming from stresses, and necrosis, representing the accidental cell death, is mainly caused by uncontrolled events [71, 72]. During 3D bioprinting, cell deaths and injuries mainly come from the stresses imposed on the living cells. There are several phenotypes of cell apoptosis including cell shrinkage and condensation of nuclei, to name a few [73]. Due to the complexity of factors triggering cell damage, it is necessary to study cell damage/death based on the molecular signaling pathways [74]. It is noted cell damage mainly comes from light irradiation stress during light-based bioprinting and shear stress during nozzle-based bioprinting [75, 76]. During light-based bioprinting such as stereolithography-based bioprinting, cell apoptosis induced by UV exposure is mediated through the activation of c-Jun NH2-terminal kinase/stress-activated protein kinase (JNK/SAPK) [77]. Shear stress applied to the cells will be firstly transformed into a biological signal subsequently inducing the activation of effector caspases [73]. The apoptosis of cells is usually triggered either through intrinsic pathway, extrinsic pathway, or both pathways [78, 79]. The intrinsic pathway refers to stress-induced caspase activation of BH3 protein and the subsequent activation of caspase 3 responding to intracellular stresses such as DNA damage, while the extrinsic pathway refers to receptor-mediated caspase activation of caspase 8 and the subsequent activation of caspase 3 responding to extracellular death receptors [80]. From both pathways, it is obvious that caspase 3 which is the most common type of caspase plays an important role in shear stress-induced cell apoptosis [81]. Before being activated, caspases exist inactively as a form of pro-caspases in normal cells. The activation of caspases demonstrates the stresses imposed on the cells have already exceeded the threshold and the cells are experiencing cell apoptosis [82]. Previous work has also reported that there is a time lag for caspase 3-initiated cell apoptosis under external loadings. However, this time lag is shortened with the increase in the magnitude of the stress [79], and cells are more likely to be quickly injured under high shear stress condition. In addition to the effect of the shear stress’s magnitude on cell damage, the duration of shear stress imposed on the cells also matters, indicating an accumulative effect of shear stress on cell damage [83].

### Cell damage in inkjet-based bioprinting

Inkjet-based bioprinting has been widely utilized for numerous tissue engineering applications due to its high printing resolution and relatively high cell viability [84]. During inkjet-based bioprinting, bioink with low viscosity is usually selected to ensure smooth jetting and avoid nozzle clogging due to the limited nozzle size [85]. Most inkjet-based bioprinting systems employ either thermal or mechanical compression to generate pressure pulses for droplet ejection [86]. Therefore, inkjet-based bioprinting can be mainly classified into thermal and piezoelectric inkjet-based bioprinting [87] with both methods relying on the generation of necessary pressure for droplet ejection [88]. Generally, cells are mainly enduring the shear stress and thermal stress if applicable during inkjet-based bioprinting. The shear stress during inkjet-based bioprinting mainly comes from two parts including the ejection and landing of the cell-laden droplets. The magnitude of the shear stress which is calculated by multiplying the viscosity of the bioink by the shear rate is dependent on both the process parameters (e.g., the nozzle size and amplitude of the imposed actuation voltage waveform) [89] and the bioink properties (e.g., the viscosity of the bioink) [90]. It has been found that the shear stress imposed on the cells when landing on the substrate is even higher than that generated during the ejection process out of the inkjet nozzle [91]. A smaller droplet impact velocity has been found to significantly improve the cell viability [92, 93], while a higher droplet impact velocity generally results in more intensive degree of deformation of the cell membrane and more cell damage [94]. In addition, the evaporation of the formed droplets has been reported to negatively affect cell viability [95]. The shear stress which originates from the rheological properties of the bioink can be maximally prohibited by selecting the biological material with shear-thinning property, thus reducing cell damage [96]. The thermal stress mainly derives from the thermal inkjet-based bioprinting process. When the nozzle is heated more than 37 °C, cell damage is correspondingly enhanced. Overall, shear stress-induced cell damage usually comes from the deformation and the enlargement of cell membrane [97]. As reported, when the increase in the size of the cell membrane exceeds 5%, cell membrane cannot maintain the intact shape and becomes stretched to rupture resulting in cell death [98]. Enduring from the shear stresses [99], cell viability is generally decreased [100] and the mis-functionality of the living cells is found [101]. Bioink should be carefully selected for suitable properties, and the operating conditions should be optimized to maximally maintain cell viability [102].

### Cell damage in extrusion-based bioprinting

Extrusion-based bioprinting has been widely involved in various tissue engineering applications due to its easy implementation, low cost, and allowance on bioink’s viscosities and concentrations [103–105]. During extrusion-based bioprinting, due to the selected bioink with high viscosity for better printability and support, a great percentage of cells endures the printing-induced cell stresses and gets injured/killed, resulting in relatively low cell viability compared to other printing techniques [60, 106]. In addition, a mechanical-driven (e.g., screw-driven) dispensing system generates a higher pressure when bioprinting the bioink with high viscosity and simultaneously kills more cells [107]. The relationship between shear stress and various printing parameters such as nozzle size and viscosity of the bioink and the effects of various printing parameters on cell viability have been comprehensively studied. Generally, the shear stress mainly comes from the printing process and the materials. From the process’s perspective, when the bioink containing the living cells is extruded out of a needle, the size of the nozzle and the operating pressure directly determine the strength of the generated shear stress [102]. It is noted that the major cause of cell damage lies in the shear stress generated during the extrusion out of the needle [76], and dispensing pressure has a more significant effect compared to the size of the nozzle [108]. Briefly, the increase in dispensing pressure and the decrease in nozzle size generally lead to the increase of shear stress and thus decrease cell viability [106]. In addition, the extrusion speed also affects the shear stress and thus further affects cell viability [109]. From the materials’ perspective, bioink with higher viscosity generally generates a higher shear stress on the cells resulting in more severe cell damage. To overcome this issue, similar to inkjet-based bioprinting, bioink with shear-thinning properties is preferred to maximally decrease the imposed shear stress on the cells and thus increase cell viability during the printing process [110]. The shear stresses coming from the printing process and the materials have joined together to generate a large shear stress on the living cells, leading to the rupture of the cell membrane and a subsequent cell damage/death in extrusion-based bioprinting process [111]. To maintain cell viability after the bioprinting process, the biological materials within the bioink should be carefully chosen, and the operating conditions should be optimized [102].

### Cell damage in laser-assisted bioprinting

Laser-assisted bioprinting, on the basis of LIFT, has drawn more attention due to its superior advantages such as high printing resolution, high cell viability, and capability to print the bioink with high viscosity with little concerns of nozzle clogging [112]. There are two major types of stress, including the shear stress generated during the jetting process and that from the materials and the thermal and radiative stresses imposed on the living cells by laser exposure during laser-assisted bioprinting, among which cell injury due to the shear stress generated during the printing process has been proved to be more severe [113, 114]. The shear stress in laser-assisted bioprinting process mainly comes from two parts, including that generated by the ejection and landing process of the cell-laden droplets and that from the materials. The magnitude of the shear stress which is calculated by multiplying the viscosity of the bioink by the shear rate depends on both the process parameters (e.g., the speed of the jet due to bubble expansion for droplet formation) and the bioink properties (e.g., the viscosity of the bioink) [115]. When there is a rapid acceleration (jetting) or deceleration (landing) during the jetting process [116], huge shear stress will be imposed on the cells, causing cell injury by damaging the membrane [117] and breaking DNA double-strand [118]. The thermal and radiative stress is due to the exposure of a laser such as UV laser at one spot where UV laser may inactivate the enzymes, denature the proteins, and damage the DNA double-strand [119], and higher laser intensity may damage more cells [72]. For example, yeast cells have been proven to be easily injured when exposed to laser lights [120]. Similar to the shear stress in inkjet-based bioprinting and extrusion-based bioprinting, bioink with higher concentration/viscosity generally generates more stress on the cells and further reduces cell viability, and that is why hydrogels with shear-thinning properties are preferred. Despite the shear stress generated over each step, previous studies have demonstrated the relatively cell-friendly printing mechanism of laser-assisted bioprinting technique by investigating post-printing stem cells’ functionality and DNA [121, 122].

### Cell damage in stereolithography-based bioprinting

Based on the curing mechanism, stereolithography-based biopprinting can be further divided into two types, including conventional stereolithography where a laser beam is exposed to solidify the photocrosslinkable materials with a point-by-point movement, and DLP, in which a digital micromirror device (DMD) is in place of a physical mask curing the whole 2D pattern upon one-time exposure [123]. Holding the advantages of high printing resolution and cell viability, stereolithography-based bioprinting has been employed in the fabrication of 3D functional tissues/organs using photocrosslinkable materials over the past few years [124]. When photocrosslinkable materials are selected for bioprinting, the presence of a suitable type of photoinitiator is needed to initiate the crosslinking process and solidify the photocrosslinkable materials under UV/visible light exposure [69]. Under UV/visible light exposure, free radicals released by the photoinitiator polymerize the photocrosslinkable materials [125], and different types of photoinitiators have been found to be sensitive to different light wavelengths [126]. Due to its nozzle-free mechanism, unlike inkjet-based bioprinting or extrusion-based bioprinting, there is almost no shear stress imposed on the living cells during the printing process, and the main cell damage comes from the radiative stress from the light source and the cytotoxicity of the photoinitiator. The thermal and radiative stress are dependent on the light source. For example, when ultraviolet radiation A (UVA) with a typical wavelength of 320–400 nm is selected, cells’ nuclear DNA may be damaged which will further contribute to genomic mutations [127]; when ultraviolet radiation B (UVB) with a typical wavelength of 290–320 nm is selected, cell apoptosis can be induced by activating the death receptor CD95 [128]; and when visible light such as blue light with a typical wavelength of 405 nm is selected, its effect on cell damage will not be as severe as the two types of UV light [129]. In addition, for stable crosslinking to address the problem of oxygen inhibition, longer UV exposure or higher UV intensity is neccessary [130], which further reduces cell viability [131]. The cytotoxicity of the photoinitiator before crosslinking is another key factor reducing cell viability through contacting the living cells. However, after the light exposure, the photoinitiator has been decomposed, losing the ability to continuously affect cell viability during the post-printing process. As reported by several researchers, even the commonly used photoinitiators such as Irgacure 2959 and lithium phenyl-2,4,6-trimethyl-benzoyl phosphinate (LAP) before crosslinking are found to keep decreasing pre-printing cell viability by contacting with the living cells, especially after long printing time [132]. For example, 0.7% (w/v) Irgacure 2959 has been reported to decrease the cell viability from 80 to 25% to 6% as the printing time increased from 30 to 45 to 60 min. In addition, LAP was found to be a more biocompatible photoinitiator compared to Irgacure 2959 especially at higher concentrations. For example, the cell viability measured at 1-hour printing time was 53% for 0.9% (w/v) LAP, while this value was reduced to almost zero for Irgacure 2959 at the same concentration [133]. These stresses including the radiative stress coming from the light sources, and the cytotoxicity of the photoinitiator should also be considered when photocrosslinkable materials such as gelatin methacrylate (GelMA) are selected during other 3D bioprinting techniques such as extrusion-based bioprinting or in a hybrid bioprinting system [62].

### Cell damage modelling

As aforementioned in the above sections, for nozzle-based bioprinting such as inkjet-based bioprinting and extrusion-based bioprinting, cell injury is mainly due to the stress generated during the ejection and landing process. One simple model is the direct correlation of cell damage percentage with shear stress using a power-law function: $CD\% = k\tau ^{a}$, where *k* and *a* are power-law coefficients, $\tau$ represents shear stress, and *CD*% represents the percentage of damaged cells [134]. A more accurate model considering the effect of shear stress exposure time on cell damage was derived as follows: $CD\%=k{(\tau -{\tau }_{0})}^{a}{t}^{b}$, where *t* represents the exposure time and *b* is another power-law coefficient [135]. During the printing process, the cell membrane may become deformed or ruptured due to the stress imposed on the living cells, and for this reason, some researchers quantified cell viability by modelling the deformation of the cell membrane or checking the permeability of cell [12, 91]. For example, the cell membrane deformation can be approximately estimated using the formula: $M = C_{0} M_{0} e^{{ - 0.26D_{0} /D_{c} \left( {\frac{{\mu _{c} }}{{\mu _{0} }}} \right)^{{ - 0.56}} }}$, where *M* represents cell deformation, *M*_0_ is a value between 0 and 1, *D*_0_ represents the droplet’s diameter, *µ*_0_ represents the viscosity, and *C*_0_ represents a fitting parameter set at 5 [136]. It was also reported that cell membrane remained intact with extension of the membrane area up to 5% and cell rupture can be observed for larger expansion [98]. Therefore, cell viability can be estimated by studying cell membrane deformation. There have been several studies focusing on cell damage during extrusion-based bioprinting. For example, Nair et al. [108] derived an in vitro quantitative model to analyze cell injury caused by the process-induced mechanical disturbances while being ejected out of a micro-scale nozzle during extrusion-based bioprinting. Two typical processing parameters including the imposed pressure and nozzle diameter were controlled to obtain cell viability data under different experimental conditions. The collected data were subsequently analyzed to derive the formulation, and the final quantitative model showing the percentage of alive cells can be expressed as: $E\left({P}_{L}\right)=0.8563+0.655{x}_{1}-0.286{x}_{2}+0.0061{x}_{1}{x}_{2}-0.76{x}_{1}^{2}+0.000352{x}_{2}^{2}$, where *E(P*_L_*)* represents the expected value of the percentage of the living cells, and *x*_1_ and *x*_2_ represent nozzle diameter and pressure, respectively. In another study presented by Blaeser et al. [65], using a straightforward fluid-dynamics model, the intrinsic shear stress could be estimated using the formula: $\frac{\partial w}{\partial t}=\frac{1}{\rho s}\left(p+\rho gh-\frac{1}{2}\rho {w}^{2}-\frac{4}{d}{w}^{n}K{\left[\frac{2(\frac{1}{n}+3)}{d}\right]}^{n}\right)$, where *K* and *n* are power-law coefficients representing the rheological properties of the polymer solution, *d* represents the diameter of the nozzle, *s* represents the length of the nozzle, and *w* represents the average drop speed. Therefore, the shear stress imposed on the living cells can be numerically solved and its induced cell injury can be estimated. For example, it was found the average cell viability of L929 mouse fibroblasts decreased from 96 to 91% to 76% when the nozzle shear stress was increased from less than 5 kPa to between 5 and 10 kPa to more than 10 kPa.

For nozzle-free based bioprinting such as laser-assisted bioprinting and stereolithography-based bioprinting, cells are also enduring the thermal and radiative stress due to the exposure of laser/UV light. Several mathematical models including power-law [137] and Gompertz [138] have been presented to model cell injuries during the laser-assisted bioprinting process. For example, a typical power-law model was developed to predict cell death during laser-assisted bioprinting where cell death in percentage can be characterized using the formula: $I={k}_{1}\times {L}^{{k}_{2}}\times {A}^{{k}_{3}}$, where *I* represents the predicted cell death, *L* represents the laser fluence in mJ·cm^−2^, *A* represents the polymer concentration, and *k*_1_, *k*_2_ and *k*_3_ represent coefficients of power-law [72]. Alternatively, the Gompertz cell death model was defined as: $I={\text{exp}}\left[-{k}_{4}\text{exp}\left(-{k}_{5}L-{k}_{6}A\right)\right]\times 100$, where *k*_4_, *k*_5_ and *k*_6_ represent Gompertz constants. In addition, it has been widely reported that cell injury during stereolithography-based bioprinting is mainly caused by UV irradiation [75]. During the printing process, UV irradiation-induced cell apoptosis is led by the activation of JNK/SAPK on the cell membrane. Unlike the modelling of cell membrane rupture that predicts cell viability, this activation of protein on the membrane suggests that the apoptosis of cells increases the difficulty to predict cell viability [77]. Recently, Xu et al. [75] presented an innovative predictive modeling approach based on an ensemble learning algorithm combining ridge regression (RR), k-nearest neighbors (KNN), random forest (RF) and neural network (NN). This data-driven approach has been proven to be capable of accurately predicting cell viability under various experimental conditions during dynamic optical projection-based stereolithography. Moreover, the significance of several process parameters including UV intensity, UV exposure time, polymer concentration and layer thickness on cell viability were evaluated and compared.

## Cell viability during 3D bioprinting

As aforementioned, living cells are subjected to different sources and magnitudes of stresses resulting in different cell viability during different bioprinting processes. For example, the cells mainly endure strong shear stress during microextrusion-based bioprinting, resulting in loss of cell viability to various extend. Maintaining high cell viability is an essential key step for retaining suitable cellular behaviors, such as the proliferation and differentiation ability, thereby ensuring the 3D-bioprinted constructs are biologically similar to native tissues/organs. In this section, the typical cell viability range of each bioprinting technique will be discussed. Several typical applications and the associated cell viabilities using different types of bioprinting techniques are summarized in Table 1 [9, 21, 44, 72, 86, 124, 139–148].

**Table 1:** Summary of typical cell viability studies

Method	Bioink	Application	Cell viability	References
Inkjet-based bioprinting	PEGDMA, human chondrocytes	Cartilage-like constructs	89%	[139]
	Sodium alginate, 3T3 fibroblasts	Zigzag tubes	82%	[140]
	Sodium alginate, 3T3 fibroblasts	Vascular-like constructs	> 90% after 1 d	[141]
	PEG/peptide, hMSCs	Cartilage-like constructs	89%	[86]
Extrusion-based bioprinting	Nanofibrillated cellulose, alginate, hNCs	Aricular constructs	70%	[44]
	Alginate sulfate, nanocellulose, chondrocytes	Miniature ears	> 85% at day 14	[142]
	Polyurethane solutions and NSCs	Nerve-like constructs	> 50% after 1 d	[143]
	Alginate, fibroblasts, smooth muscle cells	Vascular-like constructs	> 91% after 7 d	[9]
Laser-assisted bioprinting	Alginate, 3T3 fibroblasts	3D constructs	> 90%	[144]
	HUVECs, biopaper	Osseous constructs	92.4%	[72]
	3T3 fibroblasts	3D constructs	> 93.5%	[145]
	Hyaluronic acid, hiPSCs	3D constructs	> 94% with control effect	[146]
Stereolithography-based bioprinting	GelMA, PEGDA, EY, 3T3 fibroblasts	3D structure	85% for at least 5 d	[21]
	GelMA, EY, 3T3 fibroblasts	3D structure	91.5%	[124]
	GelMA, HUVECs, C3H/10T1/2 cells	Prevascularized tissues	> 85%	[147]
	GelMA, LAP, mBMSCs	Native-like tissues	95%	[148]

*PEGDMA* poly(ethylene glycol) dimethacrylate, *PEG* polyethylene glycol, *hMSCs* human mesenchymal stem cells, *hNCs* human nasal chondrocytes, *HUVECs* human umbilical vein endothelial cells, *hiPSCs* human induced pluripotent stem cells, *GelMA* gelatin methacrylate, *PEGDA* polyethylene glycol diacrylate, *EY* eosin Y, *LAP* lithium phenyl-2,4,6-trimethyl-benzoyl phosphinate, *mBMSCs* mouse bone marrow mesenchymal stem cells, *NSCs* neural stem cells, *3D* three-dimensional

### Cell viability in inkjet-based bioprinting

Cell viability during inkjet-based bioprinting is mainly affected by the shear stress and thermal stress if applicable. During printing, due to the limited nozzle size, bioink with low viscosity and concentration is selected to avoid nozzle clogging and ensure continuous jetting which at the same time reduces the shear stress and protects cells from significant injuries. It is noted that bioink with a typical viscosity range from 3 to 30 mPa·s is suitable for inkjet-based bioprinting [88] and a typical cell viability range of more than 85% can be obtained [149]. Inkjet-based bioprinting has been favored for various biomedical applications due to its relatively high cell viability and deposition accuracy. For example, Xu et al. [139] selected inkjet-based bioprinting to successfully fabricate zigzag tubes with 3T3 fibroblasts encapsulated. Cell viability was maintained at over 82% (or 93% considering the control effect) after 72-hour incubation. Vascular-like constructs were fabricated by inkjet printing of the bioink composed of sodium alginate and 3T3 fibroblasts cells [140]. More than 90% of the cells were observed to be alive after 24-hour incubation. Gao et al. [141] used modified inkjet printers to co-print PEG- and peptide-based scaffold and human mesenchymal stem cells (hMSCs) to form native bone- and cartilage-like structures. The cell viability measured 24 h after printing was around 88%. Cui et al. [86] presented an innovational approach to fabricate cartilage-like tissues by simultaneously polymerizing poly(ethylene glycol) dimethacrylate (PEGDMA) during printing, and the cell viability of human chondrocytes was improved by 26% to around 89%.

### Cell viability in extrusion-based bioprinting

Similar to inkjet-based bioprinting, extrusion-based bioprinting is also a nozzle-based bioprinting technique and its cell viability is mainly determined by the shear stress and thermal and radiative stresses if photocrosslinkable materials are selected. However, due to its larger nozzle size, bioink with a viscosity range from 30 to 6 × 10^8^ mPa·s can be extruded out of the extrusion nozzle to form filaments and form the 3D constructs as designed with a layer-by-layer approach [150]. The increase in the bioink concentration and viscosity unavoidably increase the shear stress and kill more cells. Because of the high stresses imposed on the living cells during the bio-fabrication process, extrusion-based bioprinting typically provides a low cell viability ranging from 40 to 80%. However, better printing performance with higher cell viability can also be achieved by optimizing the experimental design and operation conditions [151]. Extrusion-based bioprinting has been widely used in various biomedical applications due to its low cost, easy implementation, and large allowance for bioink concentration and viscosity. For example, Ávila et al. [44] selected extrusion-based bioprinting to fabricate auricular constructs with the bioink composed of nanofibrillated cellulose, alginate and human nasal chondrocytes (hNCs). The cell viability measured immediately after bioprinting was approximately 70%. In addition, cells were found to recover during 28 d in vitro culture, resulting in increased cell viability. Müller et al. [142] extruded the bioink composed of alginate sulfate, nanocellulose, and chondrocytes out of the nozzle with different air pressure to fabricate complex structures such as a miniature ear. Cell viabilities under all conditions were maintained at more than 85% after 14 d culture and 88% after 28 d culture. Hsieh et al. [143] utilized thermoresponsive polyurethane solutions associated with neural stem cells (NSCs) to create structures that could be transplanted to heal the damaged nervous system. Cell viability was measured to be greater than 50% at 24 h after printing. Gao et al. [9] coaxially bioprinted the bioink composed of alginate and fibroblasts and bioink composed of alginate and smooth muscle cells into vascular-like constructs with multiple fluid channels. After 7 d of incubation, more than 91% of the encapsulated cells survived.

### Cell viability in laser-assisted bioprinting

Unlike inkjet-based bioprinting and extrusion-based bioprinting, laser-assisted bioprinting relies on a nozzle-free mechanism to form cell-laden droplets, which are used as the building block for 3D-bioprinted constructs. Cell viability during laser-assisted bioprinting is mainly determined by the shear stress during jetting and landing as well as the material properties and thermal and radiative stresses. Benefiting from its nozzle-free mechanism, a large range of bioink viscosity up to 300 mPa·s can be applicable to laser-assisted bioprinting, and more than 90% cell viability can be maintained [121, 152]. Because of its high cell viability and printing resolution, laser-assisted bioprinting has been involved in multiple tissue engineering applications. For example, Kawecki et al. [144] chose laser-assisted bioprinting to bioprint HUVECs onto the biopapers enabling the fabrication of prevascularized cell-based osseous constructs for bone repair. The cell viability on osseous and stromal biopaper was measured to be 92.4% and 97.4%, respectively. Gudapati et al. [72] studied the effects of various process parameters such as laser intensity, polymer concentration, and gelation time on post-printing cell viability during laser-assisted bioprinting of 3D constructs. Surprisingly, 2-minute gelation was able to provide higher cell viability by forming a gel membrane to better protect the encapsulated cells, while 10-minute gelation reduced cell viability due to the limitation of nutrient and oxygen exchange. More than 90% of the cells can be kept alive by optimizing the process parameters. Koch et al. [145] presented a study focusing on the effect of laser-related parameters such as laser wavelength and durations on cell viability during laser-assisted bioprinting. Cell viability remained at least 93.5% at any parameter combination. Later, the same group investigated the maintenance of cellular behaviors such as cell viability, proliferation, and pluripotency during laser-assisted bioprinting of the bioink containing hyaluronic acid and human induced pluripotent stem cells (hiPSCs). Over 94% of the undifferentiated hiPSCs were able to survive the bioprinting process and maintain their pluripotency.

### Cell viability in stereolithography-based bioprinting

As a rapidly developing technique, stereolithography-based bioprinting is also a nozzle-free bioprinting technique. Cell viability during stereolithography-based bioprinting is mainly determined by the thermal and radiative stresses. Benefiting from its nozzle-free mechanism, high cell viability can usually be achieved [23]. It is noted that cell viability can exceed 90% and the printing resolution can reach 10 μm [132, 153]. Therefore, stereolithography-based bioprinting has been recently favored for the fabrication of artificial tissues/organs due to its high printing resolution and cell viability. For example, Wang et al. [21] selected stereolithography-based bioprinting to construct 3D structures using the bioink with a mixture of polyethylene glycol diacrylate (PEGDA), GelMA, eosin Y (EY) and 3T3 fibroblast cells. It was found that a minimum resolution of 50 μm can be achieved and more than 85% cell viability can be maintained for at least 5 d. Later, the same group investigated the effects of GelMA and EY concentrations on the printing performance (e.g., cell viability) when bioprinting 3D constructs [124]. Approximately 91.5% cell viability can be achieved by carefully selecting the combination of polymer and photoinitiator concentrations. Zhu et al. [147] presented an effective and efficient approach to fabricate prevascularized tissues using stereolithography-based bioprinting. The encapsulated HUVECs and C3H/10T1/2 cells within the crosslinked GelMA-based tissue constructs have reached 85% high cell viability. Recently, Zhang et al. [148] developed a cost-effective and compact stereolithography-based bioprinter to create natural tissue-like constructs using the bioink containing GelMA, LAP and mouse bone marrow mesenchymal stem cells (mBMSCs). In their study, a high cell survival rate of 95% was observed.

## Cell functionality during 3D bioprinting

High cell viability is an initial key step in successful bioprinting of constructs that are geometrically, mechanically and functionally similar to native tissues/organs. Maintenance of cell functionality is another major concern of 3D bioprinting to ultimately ensure the functionality of the 3D artificial tissues/organs during culture [154]. Therefore, researchers should not only focus on the retainment of cell viability, but also the maintenance of cell functionality such as proliferation and differentiation abilities of the living cells both during the printing and culturing processes of the 3D-printed tissue constructs [149]. More specifically, surviving cells during bioprinting are expected to attach, proliferate, differentiate, and interact with the hydrogels that mimic native ECM. When stem cells are selected for bioprinting, they are expected to maintain the potency to differentiate into different cell types under the specific guidance and perform the corresponding gene expressions following the bioprinting processes [155]. For example, under specific guidance, MSCs after the bioprinting process are still expected to have the ability to be directed into osteoblasts, adipocytes or chondroblasts during in vitro culture [156]. The impacts of various shear stresses on the functionality of the living cells, particularly on several vulnerable cell types such as stem cells have been carefully studied. In this section, several typical studies on cell functionality such as cell proliferation and differentiation abilities and the related gene expression after different bioprinting processes are briefly summarized.

Yumoto et al. [157] developed a cell-friendly and almost stress-free inkjet-based bioprinting system and found that the post-printing cells were almost undamaged by examining cell viability as well as the gene expression of mESCs. In their study, the average cell viability of the prepared samples was maintained at 90% at 48 h after printing, a significant number of cells were observed to be able to proliferate, and an RNA-seq analysis was conducted on post-printing differentiation abilities and gene expressions. The experimental results showed that inkjet-based bioprinting had little effect on cell survival, and the stem cells after bioprinting retained their differentiation ability. Narayanan et al. [158] focused on the utilization of extrusion-based bioprinting to fabricate musculoskeletal soft tissue-like constructs using the bioink with a mixture of alginate, polylactic acid nanofibers and hASCs. The experimental results showed that the nanofibers enhanced the cell proliferation ability and improved cell viability. In addition, collagen and proteoglycan were detected to demonstrate successful ECM secretion and chondrogenic differentiation. Yu et al. [159] utilized a coaxial nozzle-based bioprinting system to form tubular channels using the bioink containing alginate and cartilage progenitor cells (CPCs). In their study, even though cell death was observed due to severe shear stress imposed on the cells during the ejection process, cell recovery was found during culture. In addition, the functionality and differentiation ability were proved to be maintained by checking cartilage-associated genetic marker expression. Sorkio et al. [10] selected laser-assisted bioprinting to deposit the bioink containing hSEC-LESC/hASCs, human laminin and collagen I into corneal structures with different types. As it is a relatively biocompatible bioprinting process, cell viability measured after printing was maintained at a high level. Successful expressions such as Ki67, p63α and p40, and the migration of hASCs from the printed structure to the host tissue indicate the functionality of the stem cells after printing and demonstrate the viability of using laser-assisted bioprinting associated with human stem cells to form native corneal-like structures. Hong et al. [11] selected a DLP bioprinting system to print the bioink composed of Silk-GMA and chondrocytes. Cell viability, proliferation and differentiation abilities of the encapsulated chondrocytes were still maintained at a high level during a 4-week in vitro culture. In addition, during in vivo experiments by transplanting the printed constructs into a rabbit model with the partially defected trachea, the formation of new cartilage-like tissue constructs was observed, demonstrating its feasibility for future cartilage regeneration. Cell viability, as well as cell functionality, is extremely important for the fabrication of 3D artificial tissues/organs, to ensure the engineered tissues/organs are both anatomically and functionally similar to the native tissues/organs [56, 160].

## Methods to reduce cell damage

3D bioprinting has achieved significant advancements over the past decade, enabling the fabrication of 3D artificial tissues/organs with high precision and resolution. However, enduring various stresses during the bioprinting processes may lead to the decrease of cell viability, loss of cell functionality, and eventual mis-functionality of the 3D artificial tissues/organs. Maintaining high cell viability is critical to ensure desirable biological printing performance since only the surviving cells during the bioprinting processes have the potential to retain their pluripotency such as the proliferation and differentiation ability, and thus ensure proper functionality of the 3D-bioprinted constructs. Generally, cell viability is determined by the overall stress mainly coming from two sources including that from the materials and that from the printing process. For example, in nozzle-based bioprinting such as inkjet-based bioprinting and extrusion-based bioprinting, cells are experiencing the shear stress while being ejected out of the nozzle, and in light source-incorporated bioprinting processes such as laser-assisted bioprinting and stereolithography-based bioprinting, cells are injured mainly due to the radiative stress and shear stress. The maintenance of cell viability can originate from properly dealing with these two sources of cell injury: materials and processes.

The materials suitable for 3D bioprinting should have several attributes such as good biocompatibility and biodegradability, sufficient mechanical strength, easy crosslinking mechanism, satisfying printability, and suitable rheological properties, to name a few [161]. Currently, natural polymers are more biocompatible but lack sufficient mechanical strength to support the structure during and after printing, while synthetic hydrogels have tunable physical/mechanical properties despite their inferior biocompatibility. The appearance of interpenetrating network hydrogels (IPNs), which are formed by mixing multiple separate hydrogels, has partially solved this problem. Combining the advantages of each hydrogel, IPNs have been broadly utilized in various applications [162]. However, simultaneous 3D bioprinting of multiple types of hydrogels may increase the complexity of printing and elongate the printing time because different hydrogels may have different crosslinking mechanisms. The crosslinking mechanism of the biological materials should also be considered when selecting the biological materials. For example, when materials with a photocrosslinking mechanism such as GelMA is selected for 3D bioprinting, the light exposure time should be carefully chosen to avoid excessive cell injury/damage. In addition, chemical crosslinking has been reported to be more harmful than ionic crosslinking [163]. Therefore, materials with ionic crosslinking mechanism should be considered for higher cell viability when possible. Alternatively, it would be reasonable to consider approaches to decrease the overall stress generated during the printing process to improve cell viability. Currently, the straightforward method to reduce the stress imposed on the cells and thus increase cell viability is to optimize the process parameters. For example, optimizing the printing parameters such as in extrusion-based bioprinting has proven to help minimize the stress acting on the encapsulated living cells and increasing cell viability [164]. Better design of bioprinting system has been an alternative approach to protect the cells from injury during bioprinting and increase cell viability. For example, it was reported that modifying the shape of the nozzle from a straight nozzle to a cone shape was able to reduce the shear stress generated during the printing process and thus significantly increase cell viability [165]. In addition, as mentioned in this review, the significant effect of shear stress on cell viability has been highlighted when the cell-laden droplets are landing on the substrate in droplet-based bioprinting such as inkjet-based bioprinting and laser-assisted bioprinting. Using the substrate with low-absorptivity biomaterials such as wet Matrigel or low thermal conductivity materials such as polytetrafluoroethylene (PTFE) has shown to help increase the cell survival by providing a cushion during landing [166, 167].

The currently available choices of biocompatible materials, especially the photocrosslinkable materials are still quite limited. In addition, when photocrosslinkable materials are selected for stereolithography-based bioprinting or hybrid bioprinting, the presence of photoinitiator is necessary to initiate the photocrosslinking process. However, even the two commonly used photoinitiators such as Irgacure 2959 and LAP have been proven to be harmful to the living cells before being crosslinked, especially after long-time printing [133]. Therefore, development of the next-generation polymer/bioink with enhanced properties is necessary [168], and the next-generation biological material is expected to be superior to the available ones and better meet the criteria of bioink requirements. Alternatively, from the printing process, nozzle-free mechanisms such as laser-assisted bioprintings have been reported to be capable to provide higher cell viability compared to the nozzle-based bioprinting such as extrusion-based bioprinting, as it reduces the shear stress imposed on the living cells while being ejected out from the nozzle. Therefore, it is also reasonable to consider shifting from the nozzle-based to nozzle-free bioprinting or hybrid printing to reduce cell damage and increase cell viability [169]. Machine learning which can address several limitations of physics-based models and provide an innovational avenue to predict the printing performance might also be helpful for further reducing cell damage and thus increasing cell viability. For example, in a recent study reported by Xu et al. [75], a predictive modeling approach based on machine learning was presented to understand the effects of several processing parameters on cell viability during stereolithography-based bioprinting. In the future, machine learning is envisioned to facilitate the researchers in this field to better understand the underlying mechanisms of 3D bioprinting and improve the printing performance (e.g., cellular activities).

## Conclusions

3D bioprinting has the potential to be more involved in the military such as promptly replacing the damaged tissues and organs in the combat zone and remote areas. Even though 3D bioprinting has provided an innovative approach for drug screening and replacing damaged tissues/organs, it also generates several ethical concerns. Ethical and regulatory problems have emerged since 3D bioprinting becomes more and more involved in medicine and other engineering fields. For example, it is important to examine the main risks associated with transplanting 3D-bioprinted tissues/organs into human body [170]. Therefore, collaboration between academia, industry and hospital is necessary to amend the regulations, which will facilitate the clinical transplantation of 3D-bioprinted tissues/organs [171]. In addition, official organizations such as the International Organization of Standards (ISO) and the American Society for Testing and Materials (ASTM) should develop standards and guidelines on bioprinting procedures and test methods [171]. To conclude, this review discusses the pathways triggering cell damage, and the major factors affecting cell viability during each bioprinting process, presents the models to quantify cell damage, summarizes the typical studies on cell viability and functionality, and proposes several directions for better maintaining cell viability during 3D bioprinting processes. Concisely, as a multidisciplinary field, better printing quality of the 3D-bioprinted artificial tissues/organs with higher cell viability and functionality requires the development of more suitable biological materials, continuous improvements on the printing techniques, and better design of the printing systems.

## Data Availability

Not applicable.

## Abbreviations

3D: Three-dimensional
ASTM: American Society for Testing and Materials
CPCs: Cartilage progenitor cells
DMD: Digital micromirror device
DLP: Digital light processing
ECM: Extracellular matrix
ESCs: Embryonic stem cells
EY: Eosin Y
GelMA: Gelatin methacrylate
hASCs: Human adipose-derived stem cells
hiPSCs: Human induced pluripotent stem cells
hMSCs: Human mesenchymal stem cells
hNCs: Human nasal chondrocytes
hSEC-LESC: Human embryonic stem cell derived limbal epithelial stem cells
HUVECs: Human umbilical vein endothelial cells
IPNs: Interpenetrating network hydrogels
JNK/SAPK: c-Jun NH2-terminal kinase/stress-activated protein kinase
KNN: k-nearest neighbors
LAP: Lithium phenyl-2,4,6-trimethyl-benzoyl phosphinate
LIFT: Laser-induced forward transfer
mBMSCs: Mouse bone marrow mesenchymal stem cells
mESCs: Mouse embryonic stem cells
MSCs: Mesenchymal stem cells
Na-Alg: Sodium alginate
NN: Neural network
NSCs: Neural stem cells
ISO: International Organization of Standards
PEG: Polyethylene glycol
PEGDA: Polyethylene glycol diacrylate
PEGDMA: Poly(ethylene glycol) dimethacrylate
PVA: Polyvinyl alcohol
PTFE: Polytetrafluoroethylene
Silk-GMA: Silk fibroin-glycidyl methacrylate
RF: Random forest
RR: Ridge regression
UV: Ultraviolet
UVA: Ultraviolet radiation A
UVB: Ultraviolet radiation B
